# Computerization of a routine clinical chemistry laboratory using a Sig/net 3B series multi-user computer system

**DOI:** 10.1155/S1463924688000070

**Published:** 1988

**Authors:** Stephen P. Harrison, Robert Dugdale

**Affiliations:** 1 Department of Biochemistry Bradford Royal Infirmary Duckworth Lane Bradford 9 BD9 6RJ UK; 2 Department of Medical Physics Bradford Royal Infirmary Duckworth Lane Bradford 9 BD9 6RJ UK


Journal of Automatic Chemistry, Vol. 10, No. 1 (January–March 1988), pp. 20–24

### Computerization of a routine clinical chemistry laboratory using a Sig/net 3B series multi-user computer system

**Stephen P. Harrison**
*Department of Biochemistry, Bradford Royal Infirmary, Duckworth Lane, Bradford 9, BD9 6RJ, UK*
**and Robert Dugdale**
*Department of Medical Physics, Bradford Royal Infirmary, Duckworth Lane, Bradford 9, BD9 6RJ, UK*

### Introduction

Laboratory data processing in the clinical chemistry department at the Bradford Royal Infirmary is based on a six-user Sig/net Multi-user system. Prior to 1984 the acquisition and processing of data from a Technicon SMAC 1 biochemical analyser [1] and handling of patient information was performed using Sorcerer microcomputers as described elsewhere [2].

These microcomputers had reached the limit of their useful life, and a decision to replace them was taken. Since the installation of a total pathology system appeared to be some years away, there was a requirement for an inexpensive, yet efficient, system to cover the interim period. Additional considerations were:

(1) To achieve more efficient use of hardware resources, such as printers.

(2) To provide non-dedicated terminals for software development and word-processing.

(3) To cater for recent legislation regarding data protection of patient information.

(4) To upgrade the system from floppy to Winchester disks.

The latter consideration would not only provide greater speed of access and storage capacity but, due to the sealed environment of the Winchester disk, would avoid disk crashes due to harsh laboratory conditions. This facility, coupled with the increased processor speed, would also enable the database management package of choice, dBase II [3], to run at the optimum rate.

### Functional aspects of the system

The main functional requirements placed on the system were:

(1) To arrange for the continuous reception and storage of the serial data from the SMAC analyser.

(2) To provide a facility for reformatting and editing this raw data, coupled with the production of an optimized print-out of patient results, and to permit the selective storage of validated results.

(3) To allow independent input of patient details, and to assimilate this information with the validated results provided by the facility described above. These results were to be made available for interrogation in real time and on archive, with the provision for statistical analysis if required.

(4) To provide word-processing and software development facilities for office and laboratory staff.

(5) To allow for the storage of external quality control data, and to provide a statistical package for manipulating this and any other data.

### *Hardware and software*

The Data Dynamics Sig/net 3B computer [4] is a multi-user system based on the Z-80B processor. The system consists of a number of close-coupled microcomputer modules, or 'satellites', connected together by the Sig/net 'ring' to a 'hub' unit which is dedicated to managing the disks, printers and other high-cost shared devices. Each of the satellites, comprising a Z-80B processor and 64 Kb (kilobytes) of random access memory, is associated with a particular user and communicates directly with the hub via the ring. The configuration at Bradford consisted of a hub with a 52 megabyte Winchester disk unit and a 700 Kb floppy disk drive, together with two three-user expansion box modules. The peripherals included five terminals, four matrix printers and a daisy-wheel printer.

The operating system used with this network consists of two parts: McNos (Micro Network Operating System) and Netman (Network Manager). McNos is the operating system proper, comprising a set of file-manipulation and input/output routines and a disk paging system. Netman is a subsystem which runs under McNos and implements the logic necessary to service satellite requests. Each satellite has its own instance of McNos, tailored to its specific hardware configuration, which performs file access by communicating with the hub.

McNos has a command language interpreter, KCL (Kevin's command language) which forms the main user interface and allows macro files to be constructed. A system library directory is maintained on the hard disk for programs of a general interest, or public utility programs. Each user is assigned a unique name and password which may be changed at regular intervals to provide patient data protection.

To the user, this operating system is functionally very similar to CP/M 2.2 (control program for microprocessors), and allows all CP/M 2.2 application programs to run under it.

For the main laboratory system the software was written in assembler language or using dBase II command files. dBase II was used for off-line operations, such as data editing, archiving and file manipulation. Where data capture or real-time operations were required, assembler language programs were used to update files compatible with dBase II.

### System operation

The six processors were allocated as shown in figure 1. Five satellites were connected to terminals, with the sixth processor being dedicated to data capture from the SMAC analyser. Satellite 1 was used for input of patient information, and satellite 3 was used for editing raw data from the SMAC. Satellite 4 was used for office work, satellite 5 was used for internal and external quality control purposes and satellite 6 for result processing by the Top Grade Biochemist. The system works as follows.

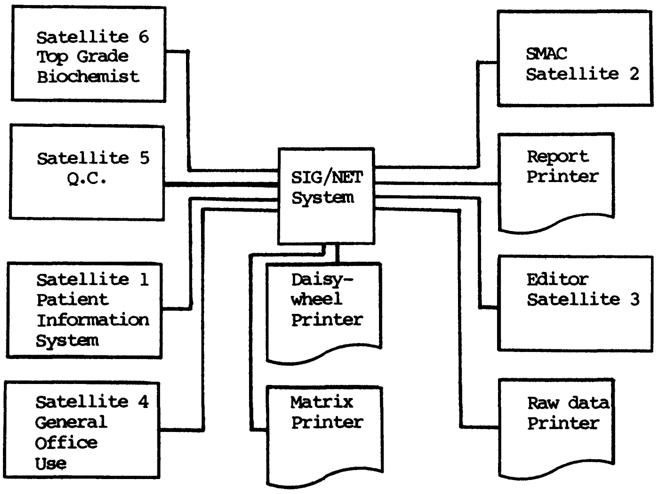

*Figure 1. Overview of data management system at the Department of Biochemistry, Bradford Royal Infirmary using a Sig/net Multi-user System.*

### *Satellite 1*

This satellite handles patient information from request forms and provides archiving facilities. Handling of current day patient information where speed of input; versatile searching and multiple checks were needed, meant that assembler had to be used. However, the archive facilities where files were not subjected to constant update, were written in dBase II. The patient entry format is depicted in figure 2. Patient entry was designed with the same order of information as the patient request form. A minimum of surname, test and identification number has to be entered to save that particular information. If any of these are absent, both audible and visual alarms are given. Age may be entered as an age in years, months, weeks or days, or as the year of birth prefixed by a 'D'. In the latter case this is converted to age in years. Consultant, hospital/ward and test fields are entered as mnemonic codes to save on storage and also to make the statistical analysis of patient request data easier. The two numbering systems in operation also have check procedures incorporated into them. It was impractical in the specimen reception system to have computer generated identification numbers and therefore the first number to be used at the beginning of the day minus 200, and the first number plus 600 are entered into the system as seed numbers. If any number is entered which is outside these limits an error is given. The same is true for any duplicate number entered into the system, and so the possibility of entering an incorrect identification number is minimized. The data is saved onto a dBase II compatible file with response time being less than a second.

```
      SURNAME [                    ]

   FIRST NAME [                ]

   AGE/D.O.B. [         ]

   CONSULTANT [    ]

      HOSP NO [         ]   HOSP/WARD [       ]

          SEX [ ]               TEST1 [    ]

        TEST2 [    ]            TEST3 [    ]

PROFILE NUMBER [      ]   BLACK NUMBER [      ]

  S=SAVE DATA [ ] R=SAVE AND HOLD

         F1 =SEARCH DAYFILE     F11 =EXIT

```

*Figure 2. Patient data entry format on user B, written in assembler.*

On pressing function key 1, a search can be made on any data field for any length of string using the same input format. For example, the system will find all patients with surname beginning with 'S', or all the Smiths etc. If the name has been entered incorrectly, the hospital number may be used, or a list of patients from a specific ward or consultant may be obtained. A search may also be made on a specific identification number. This system is linked into the validated results file and if results are wanted from the search list, the relevant number at the side of the name is input and the validated result file is scanned. If the results are present they are displayed on the screen. This means results from the SMAC may be displayed as soon as they are available and time is saved in looking to see if a specimen has been processed.

The archive result facilities were written in dBase II, for ease of software development, and because the archive files are not being constantly updated, as is the dayfile. A number of options are present, as shown in figure 3.

Entry of number seeds may be accomplished using the first option of the table. The criteria for these numbers have been mentioned previously.

```
            BIOCHEMISTRY LABORATORY DATA MANAGEMENT SYSTEM
            **********************************************

    OPTIONS :
                A = Enter number seeds
                B = Archive search
                C = Transfer patient data to archives
                D = Printout of dayfiles
                E = Edit patient information
                F = Ward counts
                G = Test counts
                Q = Exit from system

   OPTION ?  _
```

*Figure 3. Archiving and housekeeping facilities on user B, written in dBase II.*

Option B is an archive search. This may be on the hard disk or a floppy disk. The user may search on four fields: surname, hospital number and either of the two idenification numbers. The system will display the dates available on the particular disk selected and if one of these dates is input along with a surname or hospital number, the archive search will be for that particular date only. If no date is entered, the program will search the whole database and bring back all occurrences for that particular keyword. Once again the keyword can either be complete or partial but if a one letter search is made, many records will have to be scanned before the correct one is found, which may be quite time-consuming.

Option C is the option for archiving patient results at the end of the day. This option would not normally be used as the results and patient information are both archived after a hard copy is obtained.

Option D is for printing out results and patient information and then archiving this data. Patient data is printed out in alphabetical order and patient results are printed as a numerical list on the idee number. The results are archived by appending to two files, one for the validated results and the other for the patient information. Both files are re-indexed after each append operation. This is quite a lengthy procedure and may take up to three hours but gives a quick response time when searching for archived patient information. When the archive files get to a certain size, usually containing four weeks' data, the oldest week is decanted onto a floppy disk. This happens automatically when the patient data file gets to be over 12 000 records long. Option E is an editing procedure for the dayfile data in case any incorrect data has been input, for example an incorrect identification number. The position in the dayfile of the incorrect data may be located on any of four keys. The editing procedure makes use of the browse mode of dBase II. This is the so-called window into the database where 13 records are displayed at once and any item in any field may be changed.

Option F and option G both have similar functions. Option F counts up the number of patient requests for a particular ward for a single day or between two dates. Option G performs a similar function for the different tests requested to the laboratory. This produces statistical data on laboratory workloads which were done by hand before the system was implemented.

This melding of assembler and dBase II programs form the basis of the laboratory system.

*Satellite 2*

This processor is dedicated to acquisition of data from the SMAC analyser. As soon as the system powers up, the acquisition program is loaded and run and signs on at the raw data printer. It then waits for a message from the SMAC data editor on satellite 3. When this is received, the program then goes into acquisition mode. Data from the SMAC is sent serially down an RS232 line as a repeatable data string at a maximum of six streams of data per minute. The data consists of 20 result fields, a total of 256 alphanumeric characters, which are stripped of leading zeros and entered into a dBase II file ready to be processed by the SMAC editor. The dBase II file is a large file created by dBase II and filled with blanks, so there is no problem with opening new extents, which speeds up operation and simplifies programming. As the satellite is totally dedicated to result acquisition, a terminal is not required.

*Satellite 3*

This satellite is used for editing and validating raw data produced by the SMAC analyser. Most programs are written in dBase II but the main editor program contains some assembler routines where extensive string manipulation is required.

The initializing program either wipes the raw data file or continues at the next blank record. A message is passed to the SMAC acquisition program stating where it should start saving data. The acquisition program then goes into acquisition mode and the main editor program is invoked. The format of this is as in figure 4. If no data is present to be processed, the screen displays the appropriate message. However, once the SMAC begins to transmit data, the editor program will detect it by the depression of any key. Control sample results are identified by the number '666666'. The control sample results before and after the patient results are displayed

```

   Controls                 Test  Check  Test   Check              Controls seq 0101 0109  Idee  014033              Seq  0102               seq 0101 0109

   hi   low                                                         hi   low

               Sodium      139   137

  6.3L         Potassium   4.1   4.3

               Chloride    99    99     4.31   4.41   Cholest

        15H    Bicarb      28    28     234    317C   LD

               Urea        5.4   5.5    20     28     AST        45C

               Creatinine  56    65     20     23     ALT

  649*         Urate       167   150*   12     11     Bili

               ALP         85    88     28     28     GGT

               Phosphate   1.55  1.52   55     54     T Prot

               Calcium     2.12  2.13   30     30     Alb        48*   29*

  A=Accept  C=Check  E=Edit  L=Checklist  D=Delete result  P=Pack check file
  W=Clear check file  X=Edit check  Z=Printer test Y=Recall check

                        Waiting _
```

*Figure 4. SMAC data editing format on user D, written in hybrid of dBase II and assembler.*

| | | | | | | | | | |
|---|---|---|---|---|---|---|---|---|---|
| | SODIUM | 143 | mmol/L | 133-144 | LAB REF NO | 5533 | | | |
| | POTASSIUM | 4.1 | mmol/L | 3.5-5.1 | TRIGLYCERIDES | | mmol/L | 0.5-1.9 | |
| | CHLORIDE | 101 | mmol/L | 94-106 | CHOLESTEROL | 6.90 | mmol/L | 3.5-7.5 | |
| | BICARB | 22 | mmol/L | 22-31 | LD | 1062 | IU/L | 120-310 | * |
| * | UREA | 10.2 | mmol/L | 2.0-6.6 | AST | 292 | IU/L | 2-40 | * |
| * | CREAT | 173 | umol/L | 55-113 | ALT | 45 | IU/L | 2-45 | |
| * | URATE | 567 | umol/L | 120-450 | T. BILI | 8 | umol/L | 3-20 | |
| | ALP | 98 | IU/L | 30-120 | GGT | 20 | IU/L | 0-65 | |
| | PHOSPHATE | 0.86 | mmol/L | 0.7-1.4 | T. PROT | 78 | g/L | 60-80 | |
| | CALCIUM | 2.40 | mmol/l | 2.13-2.63 | ALBUMIN | 44 | g/L | 35-50 | |
| | ADJUST CA | 2.30 | mmol/L | 2.10-2.60 | GLOBULIN | 34 | g/L | 21-38 | |
| * | ANION GAP | 24.1 | mmol/L | 12-20 | RENAL INDEX | 58 | | over 90 | * |

*Figure 5. Validated results as printed out on peel-off label which is stuck to original patient request form.*

on the screen. If the result is within limits the value is not displayed, but it it is outside control limits the result is displayed along with a high or low flag. The next patient's results are displayed properly formated and if these results were being checked then the previous results are displayed alongside for comparison. These results may now be manipulated in a number of different ways. They may be edited, exchanged with the checked result, put into the check file or accepted. If accepted, the results are scanned for any error flags which have not been edited and the valid file is scanned to make sure no other results for that idee already exist. If there are none of these errors present then the results are transferred to the valid file and a hard copy is printed out on a peel-off label as in figure 5, with abnormal results appropriately indicated with asterisks. These peel-off labels are then stuck to the patient request form which is sent back to the wards. If the result was being checked then the check result is deleted from the check file. Similarly, if the results are put to check file and there is already a check present for that idee number then this is overwritten.

*Satellite 4*

This satellite is used mainly for word-processing by office staff, but also contains a dBase II program for storing and retrieving patient results for specimens sent to other laboratories.

*Satellite 5*

This satellite is used mainly for internal and external quality-control purposes. A very comprehensive statistical package (OXSTAT [5]) has been purchased for internal quality-control calculations and some external quality-control data is stored in dBase II files, although this is still under development. This satellite also contains an index of patients with paraproteinaemia which is essential for reference for our immunoglobulin work.

*Satellite 6*

This is used by the Top Grade Biochemist for clinical validation when signing reports. At present no customized software is available for this job although the software from satellite 2 is available.

### Discussion

The system was constructed with three main thoughts in mind: the present microcomputers were approaching the end of their useful lives and were prone to an increasing number of breakdowns; a large laboratory computer was some years off although a definite possibility; and the cost of the system had to be less than £8000.

The system fitted in well with current specimen reception practices and caused no upheavals when implemented. It not only replaced the aging microcomputers, but enhanced the present system by implementing a number of logical improvements. The software development team had worked in the laboratory during the previous system development and thus many problems could be both foreseen and overcome in a gradual manner. The software for each satellite was developed separately and the system was introduced in a stepwise manner so that the change-over involved the minimum of trauma. The system could be further expanded by increasing mass storage, adding more satellites to the system, or upgrading from 8 bit to 16 bit processors.

The system was written in dBase II and assembler, but all files are dBase II compatible. This provides a very flexible data structure and it is possible to transfer data to the OXSTAT statistical package, create text files accessible from word-processors and also to pass data to basic programs. The writing of our own system has put us in a much better position to assess the functional requirements of our laboratory and has made us more critical of commercial packages. Other advantages of using dBase II are that programs are written in high level language with good debug facilities and the option to link machine code programs if need be. Once data are in the database, dBase II gives the user the power to manipulate that data in almost any way possible and extract any subset of data required. This gives much scope for development in the future, although this very much depends on how long our pathology laboratory computer takes to arrive.

Problems in developing the system have been present. DBase II is not designed for use in multi-user systems, and great care regarding file security owing to multiple access by the various users was needed. Total success in this regard was not achieved and assembler programs are being designed to provide a file lock out procedure on certain files. Indexing of large files in dBase II is a lengthy procedure which must be done overnight. When this is interrupted by the occasional power failure it means files have to be re-indexed in the morning. DBase II is also not particularly good at string manipulation and the more complicated tasks take too long for real-time operation and these aspects are written in assembler.

Although the patient information and SMAC results are linked, the programs can be run separately. It is not therefore necessary to input patient information before specimens are analysed on the SMAC which means secretarial staff can plan their time more efficiently and that specimens may be analysed later in the day. Staff have generally taken well to the system because there was no great change in laboratory practice and its gradual introduction has meant that operating instructions and familiarization to the system has been leisurely. To conclude, the Sig/net multi-user computer system has fulfilled all original objectives and offers more expansion in the future if a total pathology computer for our department proves to be a pipe dream.

### References

1. Technicon Instruments Incorporated, Tarrytown, New York 10591, USA.
2. Dugdale, R., Harrison S. and Robertshaw, D. M., Data handling on a sequential multi-channel analyser computerised (SMAC): using a low-cost microcomputer. *Journal of Automatic Chemistry*, **4**, 32–34.
3. Ashton-Tate, Cofferidge Close, Stony Stratford, Milton Keynes MK11 1BY, UK.
4. Data Dynamics Ltd, Clayton Road, Hayes, Middlesex UB3 1BD, UK.
5. Wallingford Computing Services Ltd, 72 Brookmead Drive, Wallingford, Oxford OX10 9BJ, UK.

### ALTERNATIVE METHODS FOR TRACE ELEMENTS

*To be held on 10 February 1988 at the Scientific Societies Lecture Theatre, 23 Savile Row, London W1*

Organized by the *Analytical Division, Royal Society of Chemistry (Burlington House, London W1V 0BN; tel.: 01 437 8656)*, the meeting includes the following presentations:

Some aspects of ICP-MS in food analysis
*D. J. McWeeny (Food Science Laboratory, Norwich)*
Automated, continuous-flow analysis of trace metals in water using stripping chronopotentiometry
*C. M. G. van den Berg (Department of Earth Sciences, University of Liverpool)*
HPLC determination of trace metals
*Phil Jones (Department of Environmental Sciences, Plymouth Polytechnic)*
Chemical analysis at solid surfaces
*Jeremy Ness and D. J. Joiner (Surface Characterisation Section, Unilever Research, Port Sunlight Laboratory)*

### CHROMATOGRAPHY IN OCCUPATIONAL HYGIENE

To be held on 4 February 1988 at the Royal College of Surgeons at 35 Lincoln's Inn Fields, London WC2. This meeting is also being organized by the Analytical Division, Royal Society of Chemistry.

Occupational hygiene—the need for reliable analytical information—an overview
*J. Bridges (Robens Institute, University of Surrey)*
Experiences from an independent consultancy practice of the use of chromatography in questions of occupational hygiene
*D. Simpson (Analysis For Industry, Thorpe-le-Soken)*
Chromatography in occupational hygiene—the hygienist's view
*S. J. Lewes (BP International Ltd, Group Occupational Health Centre, Guildford)*
An improved method for the determination of ethylene oxide in air, using 3M's diffusion samplers
*P. E. Tindle (Shell UK Ltd, Thornton Research Centre, Chester)*
New perspectives in diffusive sampling
*R. H. Brown (Health and Safety Executive, London)*
Operator exposure to agro-chemical sprays
*B. S. Stevenson and T. McDonald (Robens Institute, University of Surrey)*
Introducing the new HSE work-place analytical scheme for proficiency (WASP) for occupational hygiene laboratories
*N. L. West (Health and Safety Executive, London)*

*Details from RSC (as above).*

### RECENT ADVANCES IN ELECTROCHEMISTRY

This is a joint half-day meeting of the Northern Ireland Region of the Analytical Division, with the Industrial Division, Northern Ireland Region and the Northern Ireland Local Section of the Royal Society of Chemistry, and will take place on Wednesday, 20 April 1988, in Room LG34, the David Keir Building, The Queen's University of Belfast at 2.00 p.m.

*Further details can be obtained from W. J. Swindall, Department of Chemistry, David Keir Building, Queen's University, Belfast BT9 5AG, UK.*

